# Coto Bark in Diarrhea of Phthisis

**Published:** 1880-06

**Authors:** 


### COTO BARK IN DIARRHEA OF PHTHISIS.

The coto bark imported from Bolivia, is employed in Germany and England, in the treatment of the diarrhea of phthisis. Burney Yeo recommends the following mixture, which is very efficacious without being disagreeable to the taste.

℞ Tinct. cotonis.
Tinct. cardamonie āā gtt lx.
Mix and add gradually
Mucilag. acacia, 12 grammes.
Syrupi simp. 8 "
Aquae 180 "

Two or three teaspoonfuls of this mixture, taken several times, suffices to arrest the severest forms of diarrhea. The bark may be given in the extract, which has the same properties.—*La France Medicale.*

